# Prevalence and associated factors with non-alcoholic fatty liver disease in a group of obese women in Tunisia

**DOI:** 10.11604/pamj.2025.51.1.46987

**Published:** 2025-05-02

**Authors:** Fatma Boukhayatia, Sana Khamassi, Farah Rezgui, Haithem Yaacoub, Haifa Abdesselem, Emna Bornaz, Kamilia Ounaissa, Saida Boumefteh, Imene Hedfi, Faika Ben Mami, Chiraz Amrouche

**Affiliations:** 1 Department of Nutritional Diseases (D), Zouhair Kallel Institute of Nutrition and Food Technology, Tunis, Tunisia,; 2 Gastroenterology Service B, Rabta Hospital, Tunis, Tunisia,; 3 Department of Nutritional Diseases (C), Zouhair Kallel Institute of Nutrition and Food Technology, Tunis, Tunisia

**Keywords:** Obesity, fatty liver disease, associated factors, nutritional intake

## Abstract

**Introduction:**

non-alcoholic fatty liver disease (NAFLD) is a leading cause of chronic liver disease, often undiagnosed, and its prevalence is closely linked to obesity and diabetes. This study aimed to screen for NAFLD in obese women and identify factors associated with both steatosis and fibrosis.

**Methods:**

a cross-sectional study was performed on 40 obese female patients. Data were collected through clinical examination, laboratory tests, anthropometric measurements, dietary survey. The assessment of NAFLD was based on the use of Fibroscan.

**Results:**

mean age was 43.56±12.75 years. The majority of the patients (65%) were inactive. The mean duration of obesity was 19.95±12.93 years, and morbid obesity was observed in 45% of the patients. Steatosis was detected in 75% of the population with 38.5% in its advanced stage, while fibrosis was identified in 90% of patients, of which 37.5% were in the advanced stage. Multivariable analysis revealed that the duration of obesity progression was positively associated with both the presence (B=0.12, 95% CI 1.00-1.26; p=0.034) and the severity (B=0.08, 95% CI 1.00-1.17; p=0.029) of steatosis. An active lifestyle was negatively associated with the severity of steatosis (aOR=0.14, 95% CI 0.02-0.80; p=0.027), while fiber deficiency intake was positively associated with the severity of fibrosis (aOR=7.04, 95% CI 1.30-37.88; p=0.023).

**Conclusion:**

these findings underscore the importance of early screening for NAFLD in this population and suggest that tailored nutritional management may be beneficial in addressing obesity-related liver conditions.

## Introduction

Non-alcoholic Fatty Liver Disease (NAFLD) refers to the accumulation of excess fat in the liver unrelated to alcohol consumption. It includes two conditions: simple steatosis and non-alcoholic steatohepatitis (NASH). The more severe form, known as steatohepatitis (NASH), is brought on by inflammation of the hepatic parenchyma and hepatocyte suffering [1]. The more severe form, NASH, is characterized by hepatic inflammation and hepatocyte injury [2].

The global obesity epidemic has led to an increased prevalence of NAFLD and NASH, making them among the most common liver diseases worldwide [3]. Indeed, over 25% of the world's population has fatty liver disease of metabolic origin, with 10% to 30% of cases potentially developing into non-alcoholic steatohepatitis (NASH) [4]. This progression poses a significant risk of developing cirrhosis, hepatocellular carcinoma, and liver failure, and is often accompanied by comorbidities such as type 2 diabetes, cardiovascular disease, and an elevated risk of extrahepatic cancers [4]. Liver biopsy, considered as the standard assessment for NASH diagnosis, is an invasive method that cannot be used as the first line of screening for NASH [5]. Consequently, non-invasive diagnostic techniques, such as blood tests and liver elastography, are recommended for assessing liver steatosis and fibrosis, especially in high-risk populations like obese patients [6]. Despite the increasing recognition of NAFLD and its potential complications, few studies have focused specifically on its prevalence and associated factors in obese women, particularly in regions such as Tunisia. This study aimed to screen for non-alcoholic fatty liver disease in obese female patients and to identify factors associated with both steatosis and fibrosis.

## Methods

**Study design and setting:** we conducted a descriptive cross-sectional study at the Department of Nutritional Diseases D, Institute of Nutrition and Food Technology, Tunisia, from August 1^st^, 2023, to October 28^th^, 2023. This study aimed to screen for NAFLD in obese women and to identify factors associated with both steatosis and fibrosis.

**Study population:** the study included 40 female patients aged between 18 and 64 years, with a BMI ≥30 kg/m^2^, as per the WHO classification [7], and who provided informed and voluntary consent. Exclusion criteria included pregnant or lactating women, patients with a history of alcohol consumption, individuals with pre-existing liver disease, and those on long-term hepatotoxic medications.

**Data collection:** each patient underwent a comprehensive investigation that covered general background information, as well as specific details regarding family history of metabolic disorders, obesity-related complications, current treatments, weight history, and investigation of dietary behavior problems. The level of physical activity was assessed using the Ricci and Gagnon auto-questionnaire provided by the French National Authority for Health for adult patients [8]. Every patient underwent a thorough physical examination, which was complemented with anthropometric measurements including weight (W), height (H), waist circumference (WC) and hip circumference (HC). Body composition was assessed using a Tanita BC 418 MA bioimpedance analyzer. Biochemical analyses included glycemic parameters, lipidic parameters, hepatic function tests and a hemogram. HOMA-IR was used to evaluate insulin resistance [8]. Dietary Intake was measured by 24-h recall method. Food intake of each subject was analyzed for energy, macronutrients, and micronutrients intake by using Nutrilog software which is based on the CIQUAL 2020 composition table validated and provided by the ANSES [9]. The assessment of NAFLD was based on the use of Fibroscan. The procedure was carried out in the gastroenterology department, using an XL probe. We also used the non-invasive FIB-4 test to evaluate fibrosis, which is considered one of the simplest and extensively validated non-invasive tests in the context of NAFLD [5].

**Definitions:** abdominal obesity was defined by a WC exceeding 80cm. Steatosis was classified into four stages based on the Controlled Attenuation Parameter (CAP) results: S0 for CAP < 294 dB/m, S1 for CAP between 295-309 dB/m, S2 for CAP between 310-330 dB/m, and S3 for CAP > 331 dB/m. The presence of advanced fibrosis was assessed using Fibroscan, with diagnostic thresholds for advanced fibrosis set at <7.9 kPa (exclude) and >9.6 kPa (confirm) [10,11]. The FIB-4 thresholds for excluding and confirming advanced fibrosis were <1.30 (exclude) and >2.67 (confirm), with a gray zone between these values where the diagnosis is uncertain [10].

**Statistical analysis:** data were analyzed using SPSS 25. Descriptive statistics are presented as means and standard deviations for continuous variables, and frequencies and proportions for categorical variables. For comparisons, the Pearson's Chi-squared test was used for categorical variables, while the independent samples t-test was used for continuous variables. A p-value of <0.05 was considered statistically significant. For multivariable analysis, we applied stepwise descending logistic regression. Initially, all factors with p-values < 0.05 and those between 0.05 and 0.15 from the univariable analysis were included in the model. At each step, the least significant factor was removed until the final model was obtained. Adjusted odds ratios were calculated to determine the independent effect of each factor. For all analyses, a two-tailed P-value of 0.05 was considered significant.

**Ethical considerations:** the study protocol was conducted under the ethical principles outlined in the Declaration of Helsinki. Before enrollment, all participants provided informed consent after being fully informed of the study's purpose. The ethical committee of the National Institute “Zouhaier Kallel” of Nutrition and Food Technology of Tunis approved this study on August 8, 2023, under reference 31/2023. This work has no conflicts of interest.

## Results

The general characteristics of the total sample are shown in Table 1. According to this study, 75% of the population had steatosis as detected by Fibroscan: 17.9% in stage S1, 17.9% in stage S2, and 38.5% in stage S3. Additionally, 90% of our patients had fibrosis with F1 in 52.5% of cases, F2 in 15% of cases, F3 in 5% of cases, and F4 in 17.5% of cases. Regarding the FIB-4 score, the majority of our patients (87.5%) had a FIB-4 score below 1.3, and none had a score higher than 2.67. Furthermore, we examined the elasticity rate for patients with a FIB-4 score greater than 1.3. These findings revealed that more than half of these patients (60%) had elasticity rates greater than 9.6 kPa. Among these patients, 20% had fibrosis grade 3 (F3), and 40% had fibrosis grade 4 (F4).

**Table 1:** general characteristics of the study population

	% or mean ± SD		
Age (years)	43.56 ± 12.75		
Socio-economic level	Low		23
	Mild		73
	High		4
Physical activity level	Inactive		65
	Active		35
	Very active		0
Duration of obesity progression (years)	19.95 ± 12.93		
BMI (kg/m^2^)	40.36 ± 7.17		
Obesity class	Class 1		12.5
	Class 2		42.5
	Class 3		45.0
WC (cm)	119.80 ± 14.61		
Body fat mass (kg)	49.89 ± 12.13		
Body muscle mass (kg)	55.23 ± 8.68		
Comorbidities	Diabetes		55
	High blood pressure		35
	Dyslipidemia		60
	Metabolic syndrome		75
	Hyperuricemia		28

BMI: body mass index; WC: waist circumference

The clinical and biological parameters of NAFLD patients in both the steatosis and fibrosis stages, as well as their nutritional profiles, are compared in Table 2, Table 3 between the affected and unaffected patients. Additionally, we performed an analysis of various clinical, biological, and nutritional factors based on the severity of steatosis and fibrosis, as illustrated in Table 4, Table 5.

**Table 2:** association between clinical and biological parameters and the presence or absence of NAFLD

	Steatosis					Fibrosis				
	Absence (n=10)	Presence (n=30)	p value	Adjusted ORs or B (95% CI)	p value	Absence (n=4)	Presence (n=36)	p value	Adjusted Ors (95% CI)	p value
Age (years)	42.44 ± 12.86	43.90 ± 12.92	0,086			50.75 ± 2.21	42.74 ± 13.21	0.28	-	-
Duration of obesity progression (years)	11 ± 8.50	22.72 ± 12.90	**0.015**	0.12 (1.00 -E1.26)	**0.034**	4.50 ± 3.53	20.81 ± 12.72	0.082	-	-
Metabolic syndrome, % (n)	10(1)	57(17)	**0.010**	-		25(1)	47 (17)	0.39	-	
Physical activity level, % (n)	30 (3)	77 (23)	0.09			75 (3)	64 (23)	0.63		
Inactive Active	70 (7)	23 (7)				25 (1)	36 (13)			
Diabetes, % (n)	60 (6)	53 (16)	0.71			0	61 (22)	**0.02**		
High blood pressure, % (n)	30 (3)	37 (11)	0.70			25 (1)	36 (13)	0.65		
Dyslipidemia, % (n)	70 (7)	57 (17)	0.45			25 (1)	64 (23)	0.13		
BMI (kg/m^2^)	36.94 ± 5.05	41.86 ± 7.4	0.059			38.85 ± 7.32	40.83 ± 7.23	0.60		
Morbid obesity, % (n)	30 (3)	53 (20)	**0.042**			50 (2)	42 (21)	0.39		
WC (cm)	112.80 ± 11.35	122.13 ± 14.987	0.08			119.75 ± 16.64	119.81 ± 14.63	0.99		
Body fat mass (kg)	43.22 ± 7.09	52.11 ± 12.72	**0.043**			46.23 ± 12.57	50.30 ± 12.20	0.53		
Body fat mass (%)	42.44 ± 6.32	47.94 ± 5.76	**0.015**			46.08 ± 6.48	46.62 ± 6.38	0.87		
Visceral fat			**0.042**					0.16		
level	70 (7)	33 (10)				75 (3)	39 (14)			
Mild, % (n)	30 (3)	67 (20)				25 (1)	61 (22)			
High, % (n)	-	-				-	-			

OR: Odds ratio; B: Coefficient of regression; BMI: body mass index; WC: waist circumference; AST: aspartate transaminase; ALT: aspartate aminotransferase; GGT: gamma glutamyl transferase; ALP: alkaline phosphatase; DB: direct bilirubin; ADW: adjusted body weight

**Table 3:** association between nutritional parameters and the presence or absence of NAFLD

	Steatosis					Fibrosis				
	Absence (n=10)	Presence (n=30)	p value	Adjusted Ors or B (95% CI)	P value	Absence (n=4)	Presence (n=36)	p value	Adjusted Ors (95% CI)	p value
Total energy intake (kcal/d)	3057.50 ± 925.585	2828.63 ± 738.24	0.43	-		3217.75 ± 731.13	2848.97 ± 789.48	0.38	-	
Proteins (g/kg of ABW/d)	1.74 ± 0.39	1.51 ± 0.43	0.40			1.74 ± 0.40	1.53 ± 0.49	0.41		
Carbohydrate (g/kg of ABW/d)	5.61 ± 2.20	4.77 ± 1.49	0.45			5.77 ± 0.63	4.89 ± 1.77	0.33		
Excess carbohydrate intake, % (n)	60 (6)	43 (13)	0.36			0	58 (21)	**0.027**		
Excess fructose intake, % (n)	30 (3)	23 (7)	0.67			25 (1)	25 (9)	1		
Fat intake (g/kg of ABW/d)	2.08 ± 0.68	2.12 ± 0.54	0.87			2.33 ± 0.92	2.08 ± 0.53	0.43		
Excess SFA intake, % (n)	10 (1)	13 (14)	0.78			0	14 (5)	0.43		
Excess MUFA intake, % (n)	50 (5)	37 (11)	0.45			75 (3)	36 (13)	0.13		
Excess PUFA intake, % (n)	100 (10)	97 (29)	0.55			100 (4)	97 (35)	0.74		
W6 intake (g)	26.02 ± 12.84	29.27 ± 10.59	0.43			38.35 ± 6.71	27.36 ± 11.02	0.060		
W6/W3 ratio	19.28 ± 8.35	21.55 ± 9.52	0.50			29.42 ± 3.01	20.04 ± 9.18	0.052		
EPA deficiency intake, % (n)	10 (1)	3 (1)	0.40			0	6 (2)	0.63		
DHA deficiency intake, % (n)	20 (2)	7 (2)	0.22			0	11 (4)	0.48		
Fiber deficiency intake, M (n)	50 (5)	67 (20)	0.34			25 (1)	67 (24)	0.10		
Zinc intake (mg/d)	10.44 ± 3.10	10.13 ± 3.71	0.81			11.42 ± 2.50	12.13 ± 3.42	0.98		
Manganese intake deficiency, % (n)	0	17 (5)	0.16			0	14 (5)	0.43		
Vitamin C deficiency intake, % (n)	50 (5)	47 (14)	0.85			0	50 (18)	0.34		
Vitamin A deficiency intake, % (n)	90 (9)	80 (24)	0.47			75 (3)	83 (30)	0.67		
Vitamin E deficiency intake, % (n)	0	7 (2)	0.40			0	6 (2)	0.63		
Selenium deficiency intake, % (n)	0	7 (2)	0.40			0	6 (2)	0.63		

OR: Odds ratio; B: Coefficient of regression; SFA: saturated fatty acid; MUFA: monounsaturated fatty acid; PUFA: polyunsaturated fatty acid; W6: linoleic fatty acid; W3: linolenic fatty acid; EPA: eicosapentaenoic acid; DHA: docosahexaenoic acid

**Table 4:** association between clinical and biological parameters and the severity of NAFLD

	Steatosis					Fibrosis				
	S < 2 (n = 17)	S ≥ 2 (n = 23)	p value	Adjusted ORs (95% CI)	p value	F < 2 (n = 25)	F ≥ 2 (n = 15)	p value	Adjusted ORs (95% CI)	p value
Age (years)	42.47 ± 11.21	45.00 ± 13.99	0.54	-		43.76 ± 12.98	44.20 ± 12.92	0.92	-	
Duration of obesity progression (years)	14.31 ± 9.19	24.05 ± 13.87	**0.02**	0.08 (1.00 -E1.17)	**0.029**	19.57 ± 13.02	20.53 ± 13.21	0.82		
Metabolic syndrome, % (n)	24(4)	61 (14)	**0.019**	-		28 (7)	73 (11)	**0.005**		
**Physical activity level, % (n)**			**0.009**	0.14 (0.02 -E0.80)	**0.027**			0.63		
Inactive	41 (7)	83 (18)				64 (16)	67 (10)			
Active	59 (10)	17 (4)				36 (9)	33 (5)			
Diabetes, % (n)	59 (10)	52 (12)	0.67	-		48 (12)	67 (10)	0.25		
High blood pressure, % (n)	29 (5)	39 (9)	0.52			28 (7)	47 (7)	0.23		
Dyslipidemia, % (n)	71 (12)	52 (12)	0.24			56 (14)	67 (10)	0.50		
BMI (kg/m^2^)	37.00 ± 5.00	43.31 ± 7.43	**0.004**			38.37 ± 4.51	44.41 ± 9.16	**0.008**		
Morbid obesity, % (n)	29 (5)	78 (18)	**0.002**			44 (11)	80 (12)	**0.026**		
WC (cm)	112.65 ±11.34	125.09 ± 14.70	**0.006**			116.72 ± 11.05	124.93 ± 18.44	0.085		
Body fat mass (kg)	43.48 ± 8.66	54.63 ± 12.30	**0.003**			46.20 ± 9.15	56.05 ± 14.20	**0.011**		
Body fat mass (%)	43.04 ± 6.97	49.17 ± 4.30	**0.001**			45.79 ± 5.57	47.86 ± 7.40	0.32		
**Visceral fat level, % (n)**			**0.03**					0.088		
Mild	41 (7)	40 (9)				40 (10)	40(6)			
High	0	30 (7)				8(2)	33(5)			
HOMA-IR	2.37 ± 2.82	3.23 ± 4.81	0.60			1.87 ± 2.77	4.93 ± 5.49	0.074		
Hypertriglyceridemia, % (n)	94 (16)	70 (16)	0.55			84 (12)	73 (11)	0.41		

OR: Odds ratio; B: Coefficient of regression; ABW: adjusted body weight; BMI: body mass index; WC: waist circumference; AST: aspartate transaminase; ALT: aspartate aminotransferase; GGT: gamma glutamyl transferase; ALP: alkaline phosphatase; DB: direct bilirubin; ABW: adjusted body weight

**Table 5:** association between nutritional parameters and the severity of NAFLD

	Steatosis					Fibrosis				
	S < 2 (n = 17)	S ≥ 2 (n = 23)	p value	Adjusted ORs (95% CI)	p value	F < 2 (n = 25)	F ≥ 2 (n = 15)	p value	Adjusted ORs (95% CI)	p value
Total energy intake (kcal/d)	2954.65 ± 761.56	2835.00 ± 811.53	0.63	-		3015.88 ± 852.15	2669.13 ± 618.36	0.18	-	
Proteins (g/kg of ABW/d)	1.59 ± 0.49	1.52 ± 0.47	0.66			1.61 ± 0.52	1.46 ± 0.40	0.36		
Carbohydrate (g/kg of ABW/d)	5.26 ± 1.67	4.78 ± 1.68	0.38			5.25 ± 1.84	4.54 ± 1.40	0.20		
Excess carbohydrate intake, % (n)	53 (9)	43 (10)	0.55			52 (13)	53 (8)	0.93		
Excess fructose intake, % (n)	24 (4)	26 (6)	0.85			20 (5)	33 (5)	0.34		
Fat intake (g/kg of ADW/d)	2.06 ± 0.61	2.15 ± 0.55	0.62			2.21 ± 0.66	1.95 ± 0.34	0.72		
Excess SFA intake, % (n)	6 (1)	17 (4)	0.27			12 (3)	13 (2)	0.90		
Excess MUFA intake, % (n)	41 (7)	39 (9)	0.89			36 (9)	47 (7)	0.50		
Excess PUFA intake, % (n)	100 (17)	96 (22)	0.38			100 (25)	93 (14)	0.19		
W6 (g/d)	26.76 ± 12.82	29.72 ± 9.77	0.41			28.51 ± 12.62	28.38 ± 8.43	0.97		
W6/W3	18.21 ± 8.77	23.03 ± 9.14	0.10			19.73 ± 8.78	23.07 ± 9.78	0.27		
EPA deficiency intake, % (n)	94 (16)	22 (96)	0.82			4 (1)	7 (1)	0.71		
DHA deficiency intake, % (n)	82 (14)	96 (22)	0.16			12 (3)	7 (1)	0.59		
Excess cholesterol intake, % (n)	65(11)	52 (12)	0.43			14 (56)	60 (9)	0.80		
Fiber deficiency intake, % (n)	59 (10)	65 (15)	0.68			12 (48)	87 (13)	**0.014**	7.04 (1.30 – 37.88)	**0.023**
Zinc (mg)	10.18 ± 2.73	10.23 ± 4.08	0.96			10.79 ± 3.65	9.22 ± 3.21	0.17	-	
Manganese intake deficiency, % (n)	0	22 (5)	**0.040**			4 (1)	24 54)	**0.036**		
Vitamin C intake deficiency, % (n)	47 (8)	48 (11)	0.09			36 (9)	67 (10)	0.060		
Vitamin A intake deficiency, % (n)	94 (16)	74 (17)	0.096			84 (21)	80 (12)	0.74		
Vitamin E intake deficiency, % (n)	0	9 (2)	0.21			0	13 (2)	0.061		
Selenium intake deficiency, % (n)	0	9 (2)	0.21			4 (1)	7 (1)	0.70		

OR: Odds ratio; B: Coefficient of regression; ABW: adjusted body weight; SFA: saturated fatty acid; MUFA: monounsaturated fatty acid; PUFA: polyunsaturated fatty acid; W6: linoleic fatty acid; W3: linolenic fatty acid; EPA: eicosapentaenoic acid; DHA: docosahexaenoic acid; TEI: total energy intake

## Discussion

Although many studies have been carried out to assess NAFLD in obese patients worldwide, in Tunisia they are few. The current study aimed to screen for NAFLD in obese women and identify factors associated with both steatosis and fibrosis. Our study revealed steatosis in 75% of the study population with 38.5% in its advanced stage, while fibrosis was identified in 90% of patients, of which 37,5% were in the advanced stage. NAFLD is the most common liver condition, especially in Western industrialized countries. Our results were consistent with those reported by Atri *et al*. where a prevalence of steatosis around 73,6% was reported, diagnosed through abdominal ultrasound [11]. Furthermore, lower prevalence rate was reported by Li *et al*. and Kim *et al*. around 30.55% and 34%, respectively [12,13]. As for Abeysekera *et al*. who opted for Fibroscan, a prevalence of 20.7% was found [14]. The development of fibrosis is a progressive stage of steatosis, but sometimes this fibrosis can mask underlying steatosis. This was found in 25% of our patients who had fibrosis without visualized steatosis on Fibroscan.

The pathophysiological mechanism of developing NAFLD in obese individuals is complex. Particularly in case of associated insulin resistance, the increased flow of fatty acids to the liver is linked, on one hand, to a mass effect explained by the increase in fat mass and android fat distribution, and on the other hand, to increased lipolysis related to a reduced anti-lipolytic effect of insulin. Moreover, excess adipose tissue, especially visceral, is responsible for an increased secretion of adipocytokines, particularly TNFα, which could play a significant role in the development of NASH by exacerbating insulin resistance [4].

Our study revealed that 65% of the study population were inactive, of which 88% had steatosis. Sedentary behavior was not significantly associated with the presence of NAFLD (p=0.09) but rather with the severity of steatosis (p=0.009). Multivariable analysis showed that an active lifestyle was negatively associated with the severity of steatosis (aOR=0.14, 95% CI 0.02-0.80; p=0.027). Indeed, the positive relationship between sedentary time and NAFLD may be attributed to a higher calorie intake [15]. These same findings were reported in the investigation by Ryu *et al*. where patients who reported a resting time of 10 hours per day were more likely to have a higher total calorie intake than those who reported < 5 hours per day [16]. Moreover, the associations between resting periods and NAFLD were statistically significant.

BMI has been found to have the highest predictive value for liver steatosis. It has been demonstrated that the risk of steatosis and NASH increases with the severity of obesity [2]. The prevalence of NAFLD can reach 98% in individuals with severe obesity, particularly abdominal obesity, of which approximately 37% are in the NASH stage [17]. Bedogni *et al*. showed that an increase in BMI is a risk factor for developing NAFLD (OR = 0.26; p=0.001) [18]. These findings were consistent with the present study.

Moreover, our entire population had abdominal obesity, with waist circumference ranging from 98 cm to 160 cm. In 58% of patients, there was excess visceral fat confirmed by impedance measurement, which was associated with the presence of steatosis (p=0.042) and its severity (p=0.03) as well as the severity of fibrosis (p=0.011). These findings align with those published by Parente *et al*. [19]. The relationship between NAFLD and metabolic diseases occurs through insulin resistance. Indeed, insulin resistance is a pathological process frequently associated with NAFLD, which is a dysmetabolic condition that develops within the context of insulin resistance [20]. This relationship is strong and complex, and the preponderance of one over the other is the subject of many discussions. According to Marchesini *et al*. the HOMA-IR values were elevated in most patients with NAFLD, but the increase in HOMA score values was associated with an increased risk of metabolic syndrome, thus supporting a pathogenic role [21]. The results of this study align with ours, where the mean HOMA score was 3.25±4.32 in patients with NAFLD compared to 1.24±1.80 in healthy patients. However, the difference was not significant enough to consider it as an associated factor with steatosis.

In the present study, 45% of patients had metabolic syndrome of which 94% had NAFLD. The presence of metabolic syndrome was associated with steatosis (p=0.01). Similar to our findings, Hamaguchi *et al*. suggested that both male and female patients who met the criteria for metabolic syndrome at the beginning of their study were more likely to develop the disease [22]. Moreover, our study revealed an association between metabolic syndrome and the severity of steatosis on one hand (p=0.019) and fibrosis on the other hand (p=0.005). As a matter of fact, NAFLD is recognized as the hepatic component of metabolic syndrome, as both pathologies share insulin resistance as a common pathophysiological mechanism. EASL recommendations indicate that the search for NAFLD through liver enzymes and/or ultrasound should be part of the routine assessment in obese patients or those with metabolic syndrome [5].

The results of a meta-analysis published in 2016 by Younossi *et al*. revealed that the prevalence of type 2 diabetes was 23% and 43% for NAFLD and NASH, respectively [3]. Our study found that nearly half of the patients with NAFLD were diabetic (53%). However, diabetes was significantly associated with fibrosis only (p=0.002). The EASL recommends that in type 2 diabetic patients, the presence of NAFLD should be screened regardless of the level of liver enzymes, as these patients are at risk of progressive liver disease [5].

Data on macronutrient intake revealed that total carbohydrate intake, excess fructose or sucrose intake were not significantly associated with the presence of steatosis. However, high carbohydrate intake was associated with fibrosis (p=0.027). Nevertheless, there is a close relationship between carbohydrate intake and steatosis widely documented in the literature. It is known that a diet high in refined carbohydrates in the context of excessive caloric intake results in the deposition of intrahepatic triglycerides [23]. However, under isocaloric conditions, it is the type of carbohydrates ingested that is more relevant [24]. It has been demonstrated that a diet very high in fructose, even with a low glycemic index, can lead to insulin resistance and liver steatosis [25]. Epidemiological studies demonstrate a strong link between high fructose consumption and the incidence of NAFLD [26]. Controlled studies showed that insulin sensitivity and intrahepatic triglycerides are compromised when fructose intake exceeds 25% of energy requirements [27].

The health benefits of a fiber-rich diet, and more recently prebiotics, have been widely studied and globally accepted. More recently, their use has been linked to the improvement of several metabolic diseases, including NAFLD. Our study revealed that fiber deficiency intake was significantly associated with the severity of fibrosis (aOR=7.04, 95% CI 1.30-37.88; p=0.023). A study conducted by Zhu *et al*. demonstrated that increased dietary fiber intake was associated with a lower risk of NAFLD [28]. This study suggests that the risk of NAFLD gradually decreases with a fiber intake of 34 g/1000 kcal per day.

Data on micronutrient intake revealed that only deficiency in manganese intake was associated with both the severity of steatosis (p=0.04) and fibrosis (p=0.036). This relationship has received less attention in the literature compared to the associations with other antioxidant intakes. Current studies carried out in this direction have focused on blood manganese levels and their association with NAFLD reporting however conflicting results [29,30].

Overall, this study has several strengths and limitations, which were considered when interpreting the findings. It is one of the few studies in Tunisia to assess both steatosis and fibrosis in the obese population using the precise and validated Fibroscan tool. Additionally, our study provides valuable data on the impact of dietary intake on steatosis and fibrosis, as well as their severity. However, a main limitation is the relatively small sample size. Another limitation is that some patients declined to participate due to the inconvenience of the displacement between the two facilities for the Fibroscan procedure.

## Conclusion

NAFLD is a growing global health issue, particularly in obese populations, and remains underdiagnosed in Tunisia. Our study found a significant occurrence of both steatosis and fibrosis in obese women, with a notable association between lifestyle factors, such as physical activity, and the severity of steatosis. We also identified fiber deficiency as a key factor in the severity of fibrosis. These findings emphasize the importance of early screening for NAFLD, particularly using tools like Fibroscan, to enable early diagnosis and targeted nutritional management.

### What is known about this topic

- NAFLD is the most prevalent liver disease globally, with the highest prevalence in the Middle East and South America;
- NAFLD is strongly associated with obesity, insulin resistance, metabolic syndrome, and diabetes;
- Fibroscan is increasingly used to assess liver steatosis and fibrosis, offering greater sensitivity in early-stage liver damage detection than traditional tests.

### What this study adds

- This study reveals a significant occurrence of NAFLD in a sample of obese female patients in Tunisia, a relatively under-researched population;
- The potential role of physical activity in the progression of steatosis;
- It demonstrates a significant association between fiber deficiency and the severity of fibrosis in NAFLD, underscoring the potential role of dietary fiber in liver disease progression.
